# Online Modules to Alleviate Burnout and Related Symptoms Among Interdisciplinary Staff in Long-Term Care: A Pre-post Feasibility Study

**DOI:** 10.1177/10499091231174448

**Published:** 2023-05-10

**Authors:** Joseph H. Puyat, Karen Pott, Anne Leclerc, Annes Song, You Na Choi, Kit Chan, Chris Bernard, Patricia Rodney

**Affiliations:** 1 School of Population and Public Health, Faculty of Medicine, 8166 The University of British Columbia, Vancouver, BC, Canada; 2 Centre for Health Evaluation and Outcome Sciences, Providence Health Care Research Institute, Vancouver, BC, Canada; 3 102794 Providence Health Care, Vancouver, BC, Canada; 4 Health Sciences Association of British Columbia, Vancouver, BC, Canada; 5 Canadian Association for Spiritual Care, Toronto, ON, Canada; 6 School of Nursing, 8166 University of British Columbia, Vancouver, BC, Canada

**Keywords:** burnout, compassion fatigue, long-term care, feasibility study, ProQOL, interdisciplinary staff

## Abstract

**Background:**

The rising trend of providing palliative care to residents in Canadian long-term care facilities places additional demands on care staff, increasing their risk of burnout. Interventions and strategies to alleviate burnout are needed to reduce its impact on quality of patient care and overall functioning of healthcare organizations.

**Aim:**

To examine the feasibility of implementing online modules with the primary goal of determining recruitment and retention rates, completion time and satisfaction with the modules. A secondary goal was to describe changes in burnout and related symptoms associated with completing the modules.

**Setting:**

This single-arm, nonrandomized feasibility study was conducted in five long-term care sites of a publicly-funded healthcare organization in Vancouver, British Columbia, Canada. Eligible participants were clinical staff who worked at least 1 day per month.

**Results:**

A total of 103 study participants consented to participate, 31 (30.1%) of whom were lost to follow-up. Of the remaining 72 participants, 64 (88.9%) completed the modules and all questionnaires. Most participants completed the modules in an hour (89%) and found them easy to understand (98%), engaging (84%), and useful (89%). Mean scores on burnout and secondary traumatic stress decreased by .9 (95% CI: .1-1.8; d = .3) and 1.4 (95% CI: .4-2.4; d = .4), respectively; mean scores on compassion satisfaction were virtually unchanged.

**Conclusions:**

Modules that teach strategies to reduce burnout among staff in long-term care are feasible to deliver and have the potential to reduce burnout and related symptoms. Randomized controlled trials are needed to assess effectiveness and longer-term impact.

Burnout is a psychological syndrome characterized by feelings of energy depletion, mental detachment, and, feelings of incompetence at one’s job.^1^ Although prevalence estimates vary widely, burnout is more common among healthcare professionals.^2,3^ Risk factors for burnout include demographic characteristics, such as female sex and younger age; and, professional and clinical practice characteristics like unsupportive leadership, heavy workload, lack of job autonomy, and poor work-life balance.^4^

Burnout is also prevalent in long-term and palliative care settings. One systematic review estimated that 3% to 66% of palliative care providers experience burnout,^5^ while a 2016 study reported that 46.3% of interdisciplinary staff in long-term care settings experienced at least 1 burnout symptom.^6^ Demanding workload, time pressure, increasing complexity of healthcare demands, dealing with death and dying of patients, and, delivering bad news were identified as risk factors for burnout in palliative and long-term care settings.^7-9^

These risk factors have increasingly become a feature of Canadian long-term care as the rising trend of providing palliative care in long-term care continues to place additional demands on care staff. The trend is largely driven by a rapid increase in the population of individuals 85 years of age and over,^10^ residents’ tendency to move in after experiencing significant deterioration in health,^8^ and an increase in the proportion of residents with life-limiting conditions like cardiovascular and pulmonary diseases and dementia.^11^ About 24% of residents who move in have less than 6 months to live;^9^ the rest stay for about 2 years or until end of life, requiring support with symptom management, care planning, counselling and palliative care. In 2016-2017, around 30 000 deaths occurred in long-term care, representing 17% of all deaths recorded in Canadian provinces where data are available.

Burnout affects the well-being of the individual staff, the quality of patient care and the overall functioning of healthcare organizations. At the individual level, burnout is associated with increased risk for substance abuse, interpersonal conflicts, depression, anxiety, suicide ideation, and decline in overall health.^3,12^ Regarding patient care, healthcare providers’ burnout is linked to suboptimal care and medical errors.^5,13-16^ At the organizational level, burnout contributes to absenteeism, poor job performance, increased staff turnover, and reduced job commitment.^3,17,18^

Self-care strategies that have been identified to improve coping and resiliency include, relaxation techniques, mindfulness, meditation and self-reflective exercises.^5,19-21^ Skills-based programs were also found to be helpful like those that improve communication skills between staff and patients and their families through active listening techniques and motivational interviewing.^22-24^ Other strategies implemented at the organizational level include the creation of staff support groups, provision of telephone supports, individual mental health supports and services, staff resilience training, daily communication, leadership support, online psychoeducation, provision of respite spaces, and professional development days.^25,26^

To contribute to the growing literature on strategies that could reduce burnout among healthcare professionals in long-term care, we conducted this study aimed at examining the feasibility of implementing online modules. Our primary goal was to determine participant recruitment and retention rates, completion time and participants’ satisfaction with the modules. A secondary goal was to describe changes in burnout and related symptoms associated with completing the modules.

## Methods

### Study Design and Setting

This was a single-arm, pre-post feasibility study conducted in five long-term care sites of a publicly funded healthcare organization in Vancouver, British Columbia, Canada. Data collection was done at baseline and 4 weeks post-intervention. The study team includes current and former staff working closely with the healthcare organization’s leadership.

### Participants

Eligible participants were clinical staff (see Table 1) working at least 1 day per month (to ensure only staff with sufficient and current workplace experience participate). Recruitment was directed toward all eligible staff to determine the number and characteristics of participants that can be recruited during the study period; hence, no sample size calculation was conducted. Participants provided informed consent as per ethics approval from The University of British Columbia Research Ethics Board (#H21-01129).

**Table 1.** Distribution of Study Participants.

Characteristics	Baseline		Completed the Modules	
	n = 103		n = 64	
	Count	%	Count	%
*Sex/Gender*				
Women	87	84.5	54	84.4
Men	14	13.6	9	14.1
Prefer not to say	2	1.9	1	1.6
*Age*				
18-25	5	4.9	3	4.7
26-35	23	22.3	17	26.6
36-45	19	18.4	12	18.8
46-55	26	25.2	18	28.1
More than 55	25	24.3	11	17.2
Prefer not to say	5	4.9	3	4.7
*Job category*				
ACW, RA, UC, AW, HCW	12	11.7	9	14.1
RD, MD, MT, OT, PT, RPh, SLP, SW	16	15.5	12	18.8
Nurse	21	20.4	15	23.4
Resident care aide	49	47.6	23	35.9
Spiritual health practitioner	4	3.9	4	6.2
Prefer not to say	1	1	1	1.6
*Length of employment (years)*				
<1 year	12	11.7	8	12.5
1-5 years	33	32.0	26	40.6
6-10 years	14	13.6	7	10.9
11-15 years	15	14.6	8	12.5
More than 15 years	25	24.3	14	21.9
Prefer not to say	4	3.9	1	1.6
*Job status*				
Casual	6	5.8	1	1.6
Full time	71	68.9	45	70.3
Part time	25	24.3	18	28.1
Prefer not to say	1	1	-	-
*Multiple sites*				
Yes	31	30.1	22	34.4
No	72	69.9	42	65.6
*Number of residents regularly cared for that died in the past 6 months*				
0	2	1.9	1	1.6
1-5	33	32	20	31.2
6-10	41	39.8	24	37.5
11-15	10	9.7	8	12.5
16-20	5	4.9	4	6.2
21 or more	8	7.8	5	7.8
Prefer not to say	4	3.9	2	3.1
*Religious beliefs and/or a personal spirituality used to de-stress when feeling emotionally drained*				
Yes	65	63.1	35	54.7
No	32	31.1	24	37.5
Prefer not to say	6	5.8	5	7.8

ACW, RA, UC, AW, HCW: Arts and Crafts Worker, Rehabilitation Assistant, Unit Coordinator, Activity Worker, Health Care Support Worker.

RD, MD, MT, OT, PT, RPh, SLP, SW: Dietitian, Physician, Music Therapist, Occupational Therapist, Physical Therapist, Pharmacist, Speech and Language Pathologist, Social Worker.

### Recruitment

On January 24, 2022, we requested the communications department to send a cover letter to the work email addresses of about 700 clinical staff in five long-term care sites. The letter contained a description of the research project, a consent form, and a link to the baseline questionnaire. Recruitment posters were posted through the healthcare organization’s intranet and the study team’s website.

With the onset of the Covid-19 Omicron wave, we extended the recruitment deadline from February 18 to March 31, and advertised this extension through the intranet and printed posters containing QR codes and shortened hypertext links (bit.ly). We moved the deadline 1 final time to April 30, for a total recruitment period of 97 days. We promoted all recruitment extensions to site leaders, senior leadership and staff through emails, Zoom meetings, and in-person drop-in sessions.

### Baseline and Post-intervention Assessment

We used the online Qualtrics platform to collect baseline and post-intervention data. Participants accessed the link to the baseline questionnaire after reading the cover letter and consent form. The baseline questionnaire contained a scale for measuring burnout and close-ended questions about age, gender, job title, years of work experience in long-term care, full-time employment status, working in multiple sites, estimated number of residents regularly cared for that died in past 6 months, and whether or not participants used religious beliefs and/or a personal spirituality to de-stress.

We setup the Qualtrics platform to send automated reminders to access the modules at 2 and 4 weeks after completing the baseline questionnaire. The 4-week reminder contained a link to the post-intervention questionnaire, which could be filled out without completing the modules (to collect data on barriers to completing the modules), although we encouraged all participants to complete the modules first. We also sent several automated reminders to participants who had not yet submitted the post-intervention questionnaire, and a final reminder sent from the email address of a team member.

The post-intervention questionnaire contained the same burnout scale used at baseline and a set of questions about participants’ experience with the module, such as whether they completed the entire modules, the length of time it took to complete them, and whether they found the content satisfactory, engaging, easy to understand, and useful. Following completion of the post-intervention questionnaire, participants were provided with a CAD$30.00 honorarium.

### Intervention

The intervention consists of 2 modules (see Online Supplement for samples) delivered through an online platform for classes and training services (learninghub.phsa.ca; course number 26536) used by health administrative regions in British Columbia. Participants received the link to the intervention after completing the baseline questionnaire. Study sites provided participants with 1 hour of paid time to complete the modules. The modules are accessible free-of-charge.

The first module focuses on self-care challenges and strategies for the **INVIVIDUAL** at home and in the workplace. These strategies include self-care; nurturing resilience; cultivating self-awareness and mindfulness; saying goodbye and allowing time to grieve, debrief and reflect. The second module focuses on **TEAM** challenges and specific strategies that can be implemented in the workplace. These strategies include nurturing team relationships; improving team communication; strengthening team support; building and nurturing relationships with residents and their families; fostering effective communication with residents and their families; and, supporting families and residents at end-of-life.

We developed the modules, named **HOPE** (**H**elping **O**ptimize our **P**eople **E**nergy), based on inputs from experts in the fields of relationship-based practice, team wellness, end-of-life/palliative care, and our previous study findings.^6,8^ Texts, emotionally evocative images, participant quotes from previous studies,^6,8^ and audio recordings of team members conversing with experts were incorporated into the modules to highlight and enhance key messages.

When completing the modules, participants were invited to engage in self-reflection, recovery and learning. The modules acknowledge the changes and challenges related to working in LTC, as well as provide strategies and tools for staff to use, with the ultimate aim of reducing the risk of burnout. Participants were also provided with exercises, tools and resources that could be downloaded and printed.

### Primary Outcome Measures

We used the Professional Quality of Life Scale or ProQOL^27^ Version 5, with copyright permission. Widely used among medical and allied health professions, ProQOL contains scales measuring compassion satisfaction, burnout and secondary traumatic stress. Compassion satisfaction pertain to positive feelings associated with one’s job, whereas burnout and secondary traumatic stress refer to the negative aspects of providing care. A defining feature of burnout is the feeling of hopelessness and difficulty in effectively managing one’s work responsibilities,^27^ while fear, sleep difficulties, intrusive images, or avoidant behaviors are symptoms typically associated with secondary traumatic stress.^27^ ProQOL developers reported internal consistency reliability coefficients (Cronbach’s Alpha) of .88, .75, and .81 for compassion satisfaction, burnout, and secondary traumatic stress.^27^

### Statistical Analysis

We used descriptive statistics to characterize the study participants. To determine feasibility, we calculated and reported percentages for recruitment and retention rates, completion time, acceptability, and perceived usefulness of the modules.

We used paired t-tests to calculate the average change in pre-post scores on compassion satisfaction, burnout, and secondary traumatic stress. We also calculated absolute standardized differences to help interpret the change in scores using the widely accepted qualitative labels for interpretation: .2 (small), .5 (moderate), and .8 (large).^28^

To explore group differences in the mean change in scores associated with completion of the modules, we performed analyses stratified by demographic (e.g., age, gender) and work characteristics (e.g., occupational group, years of work experience). The results of these descriptive and exploratory analyses were summarized in a graph to provide an overall picture of the range of potential changes that can be associated with completing the modules.

## Results

**Figure 1.**
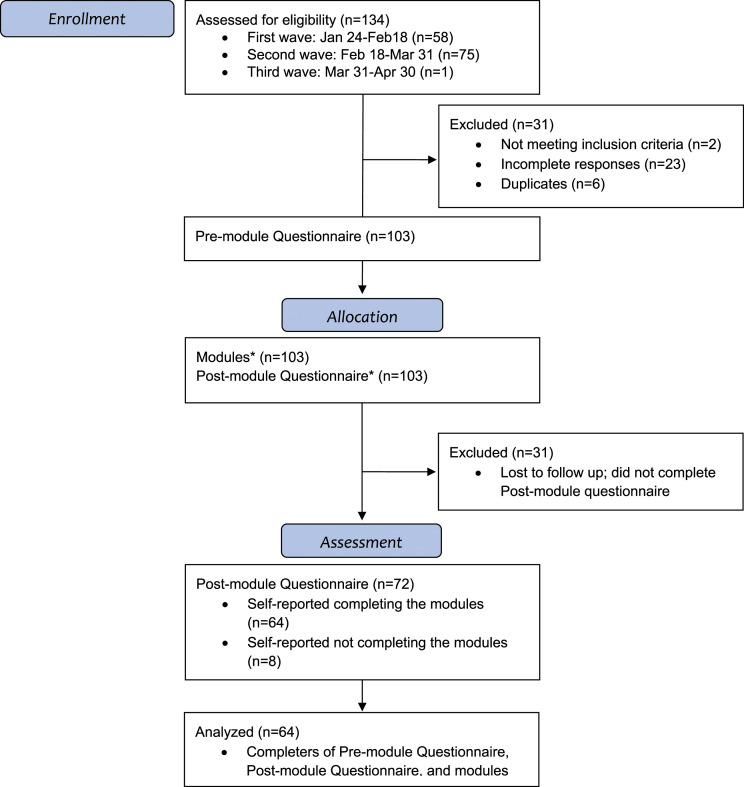
CONSORT diagram.^30,31^ *Link to the modules and Post-intervention questionnaire was sent to 103 participants who completed Pre-intervention questionnaire. Follow up reminder emails were sent 2 weeks and 4 weeks after completion of Pre-intervention questionnaire.

A total of 103 staff consented to participate, most of whom joined during the second recruitment wave. Of the 103 participants who filled the pre-intervention questionnaire, 31 (30.1%) were lost to follow-up. Of the 72 that completed the post-intervention questionnaire, 64 (88.9%) completed the modules. Data from these 64 participants were used in all analyses (Figure 1).

The key demographic and work-related characteristics of study participants were as follows: 84.4% were women, close to half (45.3%) were over 45 years of age, 53.1% had 5 or less years of work experience, 70.3% worked as full-time staff, and 65.6% worked in a single study site (Table 1). Resident care aides (35.9%) and nurses (23.4%) were the 2 most represented job categories. Up to 26.5% had cared for 11 or more residents that died in the past 6 months and more than half (54.7%) reported having religious beliefs or personal spirituality.

Post-intervention data suggest that completion rate for the modules is high (88.9% or 64/72) with most completing within an hour (89% or 57/64). Analyses of specific post-intervention questions related to comprehensibility and usefulness indicate that the modules were highly accessible and highly valued. Almost everyone (98% or 63/64) reported that the modules were easy to understand; 92% (59/64) were satisfied with the content; and, 84% (54/64) found the modules engaging. The majority of the participants found the modules helpful, with 89% (57/64) indicating that they personally benefitted from completing the modules and 95% (61/64) reporting they would recommend the modules to others.

During data collection period, respondents had 4 weeks to apply the recommended strategies. Despite this short time period, 88% (56/64) reported they were able to apply the recommended strategies for self-care and 75% (48/64) indicated they were able to apply the team strategies at work.

### ProQOL Scores

ProQOL’s internal consistency reliability at baseline were .84 (95% CI: .77-.89), .68 (95% CI: .55-.79), and .84 (95% CI: .77-.89) for compassion satisfaction, burnout, and secondary traumatic stress. Post-intervention, the coefficients were .87 (95: CI: .82-.92), .74 (95% CI: .64-.83), .84 (95% CI: .78-.89), for compassion satisfaction, burnout, and secondary traumatic stress. Pre-post correlations were .54 (95% CI: .34-.69) for compassion satisfaction, .75 (95% CI: .62-.84) for burnout, and .77 (95% CI: .65-.85) for secondary traumatic stress.

At baseline, study participants’ mean scores on compassion (38.1 ± 4.8), burnout (25.9 ± 4.6) and secondary traumatic stress (26.4 ± 6.0) did not exceed the published critical thresholds of less than 23, greater than 41 and greater than 43 for compassion, burnout and secondary traumatic stress, respectively.^27^ Examination of the distribution of individual scores further show that subscale scores did not exceed critical thresholds for all, but 1 person whose scores on compassion satisfaction went from 22 at baseline to 27, post-intervention.

Overall, there were small to moderate changes in 2 ProQOL domain scores, post-intervention. Specifically, mean scores on burnout decreased by .9 (95% CI: .1-1.8) with a standardized difference of .3, suggesting small to moderate reductions in burnout (Table 2). Mean scores on secondary traumatic stress also decreased by 1.4 (95% CI: .4-2.4) with a standardized difference of .4, indicating small to moderate reduction in secondary traumatic stress (Table 3). Mean scores on compassion satisfaction were virtually unchanged (Table 4).

**Table 2.** Average Change in Burnout among Participants that Completed the Modules.

Participant Group	Pre-intervention Mean (SD)	Post-intervention Mean (SD)	Mean Change (95% CI)	Standardized Difference	n
*All*	25.9 (4.6)	25.0 (4.9)	**−.9 (−1.8, −.1)**	−0.3	64
*Sex/Gender*						
Female	25.5 (4.5)	24.8 (5.1)	−.7 (−1.6, .2)	−0.2	54
Male	28.8 (5.0)	26.2 (4.4)	−2.6 (−5.7, .6)	−0.6	9
*Age*						
18-45	27.0 (5.0)	26.6 (5.1)	−.4 (−1.4, .7)	−0.1	32
Over 45	24.9 (4.0)	23.3 (4.5)	**−1.6 (−3.0, −.2)**	−0.4	29
*Job category*						
Nurse	26.0 (4.3)	26.1 (5.0)	.1 (−1.7, 1.8)	0.0	15
Resident care aide	24.5 (4.7)	23.2 (5.2)	−1.3 (−2.8, .3)	−0.3	23
Medical, allied & spiritual health	27.2 (4.6)	26.0 (4.4)	−1.2 (−2.5, .2)	−0.4	26
*Length of Employment*						
≤5 years	26.2 (5.6)	25.9 (5.5)	−.3 (−1.3, .7)	−0.1	34
>5 years	25.5 (3.2)	23.8 (3.9)	**−1.7 (−3.2, −.2)**	−0.4	29
*Job status*						
Full-time	25.9 (4.6)	25.0 (4.9)	−1.0 (−2.0, .1)	−0.3	45
Part-time	25.7 (4.9)	24.8 (5.1)	−1.0 (−2.7, .8)	−0.3	18
*Working in Multiple Sites*						
Yes	27.3 (4.8)	26.1 (5.7)	−1.2 (−2.9, .4)	−0.3	22
No	25.2 (4.5)	24.5 (4.5)	−.8 (−1.8, .3)	−0.2	42
*Number of residents regularly cared for that died in the past 6 months*						
10 or less	26.3 (4.9)	25.3 (5.1)	−1.0 (−2.1, .0)	−0.3	45
11 or more	25.1 (4.3)	24.3 (4.9)	−.9 (−2.5, .7)	−0.3	17
*With religious beliefs and/or a personal spirituality used to de-stress when feeling emotionally drained*						
Yes	25.1 (3.8)	24.7 (4.6)	−.4 (−1.6, .9)	−0.1	35
No	27.5 (5.7)	26.0 (5.5)	**−1.5 (−2.9, −.1)**	−0.5	24

Medical, Allied and Spiritual Health Practitioners include Arts and Crafts Worker, Rehabilitation Assistant, Unit Coordinator, Activity Worker, Health Care Support Worker, Dietitian, Physician, Music Therapist, Occupational Therapist, Physical Therapist, Pharmacist, Speech and Language Pathologist, Social Worker, and Spiritual Health Practitioners.

Positive mean change and standardized difference indicate improvement in compassion satisfaction after completing the module. Standardized differences of .2, .5, and .8 or higher are indicative of small, moderate and large changes. Bolded numbers are statistically significant at *P* < .05.

**Table 3.** Average Change in Secondary Traumatic Stress among Participants that Completed the Modules.

Participant Group	Pre-intervention Mean (SD)	Post-intervention Mean (SD)	Mean Change (95% CI)	Standardized Difference	n
*All*	26.4 (6.0)	25.1 (5.6)	**−1.4 (−2.4, −.4)**	−0.4	64
*Sex/Gender*						
Female	26.4 (5.9)	24.8 (5.3)	**−1.7 (−2.6, −.7)**	−0.5	54
Male	27.2 (6.8)	26.9 (7.6)	−.3 (−5.0, 4.4)	−0.1	9
*Age*						
18-45	28.0 (6.0)	26.3 (6.0)	**−1.7 (−3.3, −.2)**	−0.4	32
Over 45	25.1 (5.9)	23.8 (5.2)	−1.3 (−2.6, .1)	−0.4	29
*Job category*						
Nurse	26.7 (5.5)	26.7 (5.5)	.1 (−2.5, 2.7)	0	15
Resident care aide	25.3 (5.9)	24.2 (5.0)	−1.1 (−2.4, .2)	−0.4	23
Medical, allied & spiritual health	27.3 (6.5)	24.8 (6.1)	**−2.5 (−4.1, −.8)**	−0.6	26
*Length of Employment*						
≤5 years	27.4 (6.4)	26.2 (6.3)	−1.3 (−2.8, .2)	−0.3	34
>5 years	25.2 (5.5)	23.8 (4.6)	**−1.5 (−2.8, −.1)**	−0.4	29
*Job status*						
Full-time	27.1 (6.2)	25.3 (6.0)	**−1.8 (−3.0, −.6)**	−0.4	45
Part-time	24.7 (5.6)	24.4 (4.8)	−.3 (−2.1, 1.6)	−0.1	18
*Working in Multiple Sites*						
Yes	28.3 (6.8)	26.5 (6.9)	−1.8 (−3.8, .3)	−0.4	22
No	25.5 (5.4)	24.3 (4.7)	**−1.2 (−2.3, −.1)**	−0.3	42
*Number of residents regularly cared for that died in the past 6 months*						
10 or less	26.6 (5.9)	25.2 (5.4)	**−1.4 (−2.5, −.2)**	−0.4	45
11 or more	26.2 (6.7)	24.7 (6.2)	−1.6 (−3.9, .7)	−0.4	17
*With religious beliefs and/or a personal spirituality used to de-stress when feeling emotionally drained*						
Yes	25.0 (5.0)	24.6 (5.2)	−.5 (−1.7, .8)	−0.1	35
No	28.8 (7.1)	26.7 (6.0)	**−2.1 (−3.9, −.3)**	−0.5	24

Medical, Allied and Spiritual Health Practitioners include Arts and Crafts Worker, Rehabilitation Assistant, Unit Coordinator, Activity Worker, Health Care Support Worker, Dietitian, Physician, Music Therapist, Occupational Therapist, Physical Therapist, Pharmacist, Speech and Language Pathologist, Social Worker, and Spiritual Health Practitioners.

Positive mean change and standardized difference indicate improvement in compassion satisfaction after completing the module. Standardized differences of .2, .5, and .8 or higher are indicative of small, moderate and large changes. Bolded numbers are statistically significant at *P* < .05.

**Table 4.** Average Change in Compassion Satisfaction among Participants that Completed the Modules.

Participant Group	Pre-intervention Mean (SD)	Post-intervention Mean (SD)	Mean Change (95% CI)	Standardized Difference	n
*All*	38.1 (4.8)	38.4 (5.2)	.4 (−.8, 1.6)	0.1	64
*Sex/Gender*						
Female	38.2 (4.8)	38.5 (5.4)	.3 (−.9, 1.6)	0.1	54
Male	36.6 (4.3)	37.8 (4.8)	1.2 (3.1, 5.5)	0.2	9
*Age*						
18-45	37.2 (5.2)	36.9 (5.8)	−.3 (−2.0, 1.4)	−0.1	32
Over 45	39.0 (4.1)	40.5 (3.8)	1.4 (−.4, 3.2)	0.3	29
*Job Category*						
Nurse	36.6 (3.5)	37.4 (5.5)	.8 (−1.7, 3.3)	0.2	15
Resident care aide	39.5 (5.0)	40.0 (5.3)	.5 (−1.9, 2.8)	0.1	23
Medical, allied & spiritual health	37.6 (5.0)	37.6 (4.8)	.0 (−1.8, 1.9)	0.0	26
*Length of Employment*						
≤5 years	37.4 (5.3)	36.8 (5.5)	−.6 (−2.2, .9)	−0.1	34
>5 years	38.7 (4.1)	40.2 (4.3)	1.4 (−.5, 3.3)	0.3	29
*Job Status*						
Full-time	38.3 (5.0)	38.9 (5.1)	.6 (−.8, 2.0)	0.1	45
Part-time	37.7 (4.4)	37.5 (5.5)	−.1 (−2.9, 2.6)	0.0	18
*Working in Multiple Sites*						
Yes	37.6 (5.1)	37.7 (6.7)	.0 (−2.8, 2.9)	0.0	22
No	38.3 (4.6)	38.8 (4.3)	.5 (−.7, 1.7)	0.1	42
*Number of residents regularly cared for that died in the past 6 months*						
10 or less	38.0 (4.1)	38.2 (5.3)	.2 (−1.1, 1.6)	0.1	45
11 or more	38.4 (6.5)	39.0 (5.3)	.6 (−2.3, 3.4)	0.1	17
*With religious beliefs and/or a personal spirituality used to de-stress when feeling emotionally drained*						
Yes	38.3 (3.6)	38.8 (4.8)	.4 (−1.0, 1.8)	0.1	35
No	36.9 (6.0)	37.6 (5.8)	.7 (−1.8, 3.1)	0.1	24

Medical, Allied and Spiritual Health Practitioners include Arts and Crafts Worker, Rehabilitation Assistant, Unit Coordinator, Activity Worker, Health Care Support Worker, Dietitian, Physician, Music Therapist, Occupational Therapist, Physical Therapist, Pharmacist, Speech and Language Pathologist, Social Worker, and Spiritual Health Practitioners.

Positive mean change and standardized difference indicate improvement in compassion satisfaction after completing the module. Standardized differences of .2, .5, and .8 or higher are indicative of small, moderate and large changes. None of the mean change values in compassion satisfaction were statistically significant at *P* < .05.

**Figure 2.**
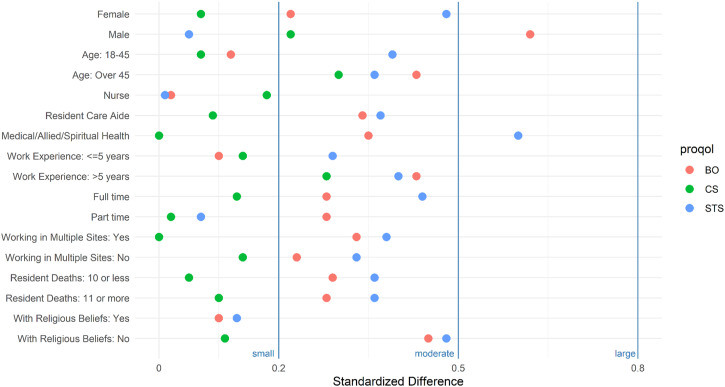
Potential Impact of Module Completion on Compassion Satisfaction (CS), Burnout (BO), and Secondary Traumatic Stress (STS). The figure summarizes the average change in mean scores (in absolute standardized difference units or Cohen’s D) by groups and ProQOL domains (Compassion Satisfaction or CS, Burnout or BO, Secondary Traumatic Stress or STS) in participants that completed the module (n = 64). Dots within the .2 and .5 areas indicate small to moderate impact, and dots within the .5 and .8 areas indicate moderate to large impact. Medical, Allied and Spiritual Health Practitioners include Arts and Crafts Worker, Rehabilitation Assistant, Unit Coordinator, Activity Worker, Health Care Support Worker, Dietitian, Physician, Music Therapist, Occupational Therapist, Physical Therapist, Pharmacist, Speech and Language Pathologist, Social Worker, and Spiritual Health Practitioners.

Results of the subgroup analyses suggest that completing the modules have small to no effect on compassion satisfaction for most occupational and demographic groups, except for those over 45 years of age and those with more than 5 years of work experience where the impact was in the moderate range (Figure 2). Results of subgroup analyses on burnout scores show small to moderate post-intervention reductions across most subgroups, except among nurses whose mean burnout scores did not change (Table 2). Interestingly, there were moderate reductions in 2 subgroups that have relatively higher burnout at baseline: males (relative to females) and those with no religious/spiritual beliefs (relative to those with such beliefs). Lastly, subgroup analyses of secondary traumatic stress scores show results similar to the subgroup analyses on burnout (Table 3). Of note are the moderate impact on other medical and allied health professionals and the moderate impact on females (relative to males) and in those that self-reported having no religious or spiritual beliefs.

## Discussion

### Main Findings

We examined the feasibility of delivering an online module to reduce burnout among interdisciplinary staff in long-term care. Results suggest the modules can be implemented online and can be completed by most in about an hour. Participants found the modules to be engaging, easy to understand, and useful. Future trials should account for a potential attrition rate of about 30% when calculating sample size. As well, future trials could benefit from having an extended recruitment period and a longer interval between outcome measurements to allow participants more time to practice the skills and strategies presented in the modules.

### Implications

We note that the recruitment and data collection period for this feasibility study occurred during the Omicron wave of the Covid-19 pandemic in Canada. Like many healthcare facilities that grappled with pandemic-related surge in infection and hospitalization, our study sites experienced staff shortages, resulting in longer hours and heavier workload for many staff. These conditions may have affected recruitment and attrition rates. It is likely that more staff would have participated had we recruited outside the pandemic period. Attrition rate could also have been lower had the delivery of the online modules been conducted after the pandemic. Despite these, however, our study team was able to collect adequate data demonstrating the feasibility of delivering the online modules in dire conditions when online resources would be most useful.

Our secondary objective was to assess changes in burnout associated with completing the modules. We found that there was a small to moderate reduction in burnout and secondary traumatic stress overall and across most demographic and occupational groups. These reductions could be due to the modules’ content being oriented primarily towards the mitigation of burnout and stress associated with working in long-term care. Additionally, the small to moderate reductions could be due to participants’ lower baseline scores on burnout and secondary traumatic stress. It is possible that greater reductions would have been observed had we recruited staff with baseline scores that are close to or exceed critical thresholds. Future trials may need to incorporate specific strategies aimed at encouraging and supporting the participation of staff with baseline scores near or beyond critical levels.

The finding about the lack of meaningful change in compassion satisfaction after module completion was not surprising given participants’ moderate scores at baseline. In this study, the mean scores in compassion satisfaction were 38.1 at baseline and 38.4 after module completion, both of which were near the higher end of the 23 to 41 moderate range.^27^ Previous studies have also found moderate levels of compassion satisfaction among health care professionals.^29^ Alternatively, it could be that our online modules lack content that could enhance compassion satisfaction.

### Strengths and Limitations

A major strength of this study was that the intervention was developed by and for long-term care staff after several years of consultation across study sites.^6,8^ The study, however, have some limitations. First, due to the absence of a control group, it is not possible to draw strong causal interpretations. Study results are most useful for gauging the acceptability and accessibility of the modules, the suitability of the outcome measurement tool, and assessing changes associated with completing the modules. Another limitation is the short interval between data collection, which precluded the assessment of beneficial effects accruing from sustained practice of the skills and strategies learned from the modules. Future trials could include longer intervals and additional follow-up periods. Third, we were unable to recruit staff with higher levels of burnout or secondary traumatic stress; our results, therefore, may have underestimated the potential impact of the modules on staff with greater needs. Finally, the sample size was small, resulting in imprecise estimates of changes that could be observed in some subgroups.

## Conclusions

It is feasible to deliver the online modules that teach strategies to reduce burnout among interdisciplinary staff in long-term care settings even during Covid-19 pandemic periods when stress and burnout levels are unusually high. The modules can be completed within an hour, are highly engaging, easy to comprehend, and helpful. Completing the modules is also associated with small to moderate decreases in burnout and secondary traumatic stress, overall, and across most demographic and occupational subgroups. An adequately powered randomized controlled trial is needed to assess effectiveness and longer-term impact.

## Supplemental Material

Supplemental Material - Online Modules to Alleviate Burnout and Related Symptoms Among Interdisciplinary Staff in Long-Term Care: A Pre-post Feasibility Study Click here for additional data file. Supplemental Material for Online Modules to Alleviate Burnout and Related Symptoms Among Interdisciplinary Staff in Long-Term Care: A Pre-post Feasibility Study by Joseph H. Puyat, Karen Pott, Anne Leclerc, Annes Song, You Na Choi, Kit Chan, Chris Bernard, and Patricia (Paddy) Rodney in American Journal of Hospice and Palliative Medicine®.
