# Supplementary material for: Online Modules to Alleviate Burnout and Related Symptoms Among Interdisciplinary Staff in Long-Term Care: A Pre-post Feasibility Study
Source: Am J Hosp Palliat Care. 2023 May 10;41(3):329–39. doi: 10.1177/10499091231174448 (PMC10802091; doi:10.1177/10499091231174448)

Online Supplement: Sample Pages from the Online Modules

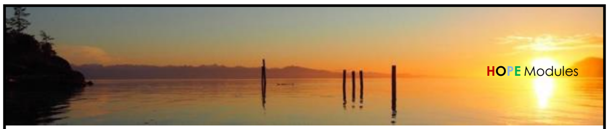

PHC LTC Research Team

Helping Optimize our People Energy

The aim of these modules is to provide interdisciplinary care staff with **tools** and **strategies** to prevent burnout and cope with stresses related to caring for dying residents.

Before entering into this period of reflection, we invite you to:

- Make yourself comfortable
- Have a cup of tea/coffee/snack, &
- Have a notepad/pen to make notes as you complete the modules
- Have access to headphones/ speaker for audio sections

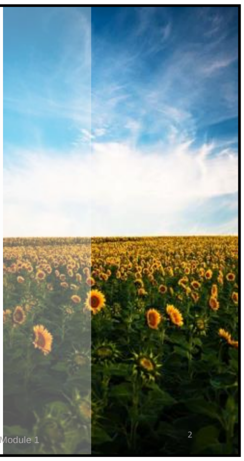

The 2016 Canadian Census showed that for the first time since records began, the number of people **aged 65** and over was *greater* than those aged 15 and under.

As the aging population has increased, more seniors are being cared for in their own homes.

This has resulted in a change in the long-term care population due to care being accessed at a **later stage** when residents have complex health conditions and a higher level of care is needed.

For many residents, care in this setting is **palliative**.

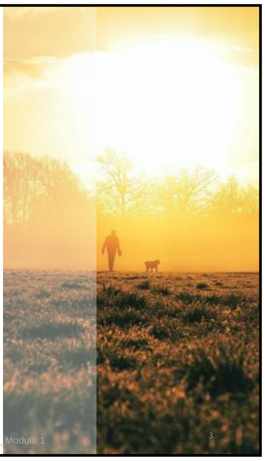

Burnout is real

Our research study exploring the stress staff experience when caring for dying residents showed that **pre COVID-19**, **nearly 50%** of PHC interdisciplinary LTC staff were experiencing **medium to high** levels of emotional exhaustion which is a risk factor for burnout.

Stress can show in different ways… Watch for any **physical, emotional** or **behavioural** signs of burnout that you may be experiencing.

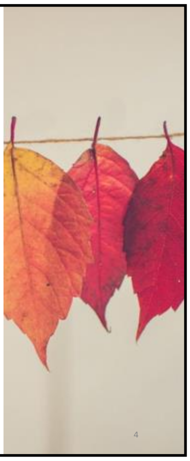

EMOTIONAL signs of **burnout**

- Sense of failure and self-doubt.
- Feeling helpless, trapped, and defeated.
- Detachment, feeling alone in the world.
- Loss of motivation.
- Cynical and negative outlook.
- Low satisfaction and sense of accomplishment.

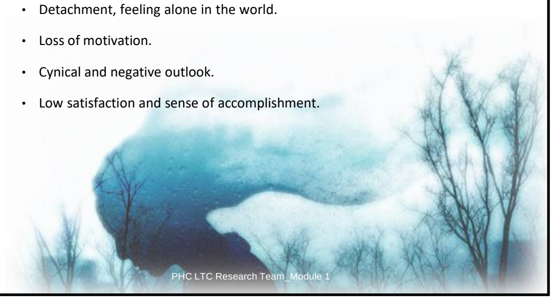

Boundaries

"**Cognitive Plexiglas**" is needed to protect ourselves from absorbing the pain and trauma of those we work with.

e.g. If caring for a dying resident who reminds us of our Mom, that we were unable to care for overseas: we might try to care for them *as if they were our Mom.* This not only causes us emotional pain, but could diminish the way in which we are focusing on the actual experience and needs of the resident. They are not our Mom.

With expressed empathy, we seek to understand the feelings, experience and behaviors of another person so we can be truly present to meet their needs.

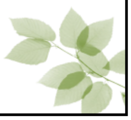

Online Supplement: Sample Pages from the Online Modules

Team cohesion is the essential ingredient of a team that functions at their best.

Cohesion is the action of forming a united whole and is comprised of a sense of **safety, inclusion** and **trust.**

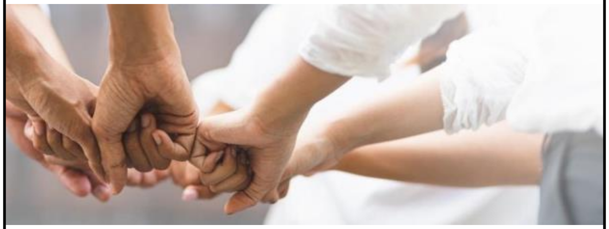

Another important team ingredient is **congruence** for individual team members:

- Can you be your authentic self?
- Is the inside of you (with regard to your own values) expressed or lived honestly to the outside world?

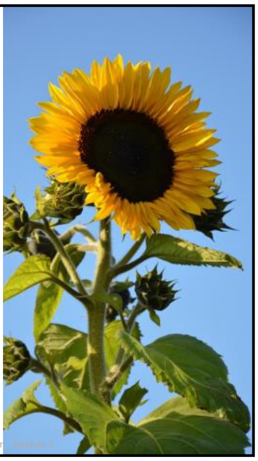

Team Relationship Strategies

Challenge	Strategy
Not being on the same page	✔ Regular communication, respectful curiosity<br>✔ Acknowledge concerns or opinions of others.
Unexpressed concern leading to build up of stress/ frustration	✔ Promote culture of safety by respecting opinions of others when different from own<br>✔ Be honest with concerns
Team members not able to be authentic selves within a team	✔ Promote inclusion and culture of acceptance and non-judgement.
Lack of trust within team	✔ Promote culture of trust by being honest with concerns. Speaking directly to those who may be causing you concern.
Low team morale	✔ Create opportunities for fun.<br>Give encouragement to colleagues.<br>Notice when colleague may be struggling and offer support and encouragement.

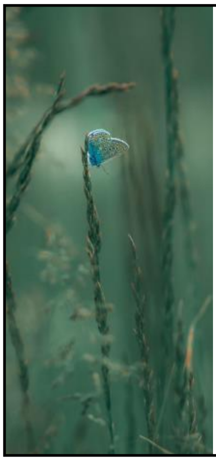

Effective Team Communication; Importance of self-awareness

- Our own experiences, values and fears will affect how we speak with others about death and dying. It's important to be aware of our own feelings about pain and suffering.
- Remember pain is what the person says it is and not what others think it ought to be. Remember how your parents responded to you when you had pain as a child? Some of those attitudes may linger in us as health care providers.

Building and nurturing **relationships** with family

Connect early and regularly with resident's family to build positive relationships and trust.

"I think it's very good to **start** with that kind of good communication then if the resident is declining, you can explain to the family and they **would trust you** because it's not one-day friendship."
(participant quote)

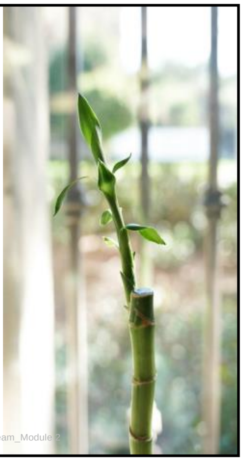

Resident comfort

"They need your touch and your care; you are needed just to be there to hold their hands, especially when there are no family members around. I can feel their hands holding me tight so I stay with them for a while."

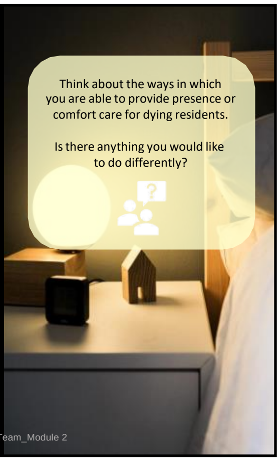

Supplement: Supplemental Material - Online Modules to Alleviate Burnout and Related Symptoms Among Interdisciplinary Staff in Long-Term Care: A Pre-post Feasibility Study [file sj-pdf-1-ajh-10.1177_10499091231174448.pdf]
